# Acute Thermal Elevation Alters Cellular Metabolic Activity, Redox Balance, and Iron Homeostasis in Butterfly Lizards (*Leiolepis belliana belliana*)

**DOI:** 10.3390/ani16172776

**Published:** 2026-09-03

**Authors:** Pawarat Chancharoen, Komsan Keawseejan, Thanakorn Intarak, Rattanatrai Chaiyasing, Prayuth Kusolrat, Worapol Aengwanich

**Affiliations:** 1 Faculty of Veterinary Sciences, Mahasarakham University, Maha Sarakham 44000, Thailand; pawarat.c@msu.ac.th (P.C.); komsan18052542@gmail.com (K.K.); thanakorn.sd425@gmail.com (T.I.); rattanatri.c@msu.ac.th (R.C.); 2 Faculty of Science and Technology, Nakhonratchasima Rajabhat University, Nakhon Ratchasima 30000, Thailand; prayuth.k@nrru.ac.th; 3 Stress and Oxidative Stress in Animal Research Unit, Mahasarakham University, Maha Sarakham 44000, Thailand

**Keywords:** oxidative stress, antioxidant defense, nitric oxide, lipid peroxidation, erythrocyte morphology, ectothermic physiology

## Abstract

Global warming is increasing the frequency of acute temperature fluctuations that may threaten ectothermic reptiles, but the underlying physiological and cellular mechanisms remain poorly understood. This study investigated physiological changes, oxidative imbalance, antioxidant systems, nitric oxide, iron metabolism, and red blood cell morphology in butterfly lizards (*Leiolepis belliana belliana*) under acute temperature elevation. Physiological responses were evaluated in vivo in butterfly lizards exposed to temperatures of 27–37 °C, whereas biochemical responses in red blood cells were examined in vitro at 27–39 °C, and red blood cell morphology was evaluated at 27–37 °C. Body temperature (*p* = 0.00000107) and pulse rate (*p* = 0.00000147) increased significantly with increasing temperature, whereas respiratory rate (*p* = 0.4185) and blood oxygen saturation (*p* = 0.7672) were not significantly affected. In blood cells, cellular metabolic activity (*p* = 0.00000674), malondialdehyde (*p* = 0.000256), protein oxidation (*p* = 0.0000526), ferric iron (*p* = 0.000000105), and vitamin E (*p* = 0.000464) increased significantly, whereas total antioxidant capacity (*p* = 0.0000000896), glutathione (*p* = 0.000453), nitric oxide (*p* = 0.000000252), and ferrous iron (*p* = 0.0000000182) decreased significantly. Hydrogen peroxide (*p* = 0.000181), glutathione peroxidase activity (*p* = 0.000000169), and catalase activity (*p* = 0.00000182) also showed significant temperature-dependent changes. Red blood cell elongation increased significantly (*p* = 0.0005), whereas nucleus size remained unchanged (nucleus length, *p* = 0.6800; nucleus width, *p* = 0.8814). Acute temperature elevation increased cellular metabolic activity and was accompanied by impaired antioxidant defense, oxidative damage, disruption of nitric oxide homeostasis, and iron dysregulation in butterfly lizard red blood cells. These findings improve our understanding of the cellular mechanisms underlying acute heat stress in ectothermic reptiles.

## Simple Summary

Rising global temperatures associated with climate change may threaten the health and survival of reptiles, whose body temperature depends directly on the surrounding environment. However, little is known about how sudden increases in temperature affect reptiles at both the whole-animal and cellular levels. This study investigated the effects of acute heat exposure on butterfly lizards by examining physiological responses and changes in their blood cells under increasing temperatures. We found that elevated temperatures increased body temperature, pulse rate, and cellular metabolic activity while reducing the natural ability of cells to protect themselves against harmful molecules produced during normal metabolism. Heat also disrupted intracellular iron balance and caused damage to lipids and proteins, together with changes in red blood cell shape. These findings suggest that sudden heat exposure can disturb normal cellular function and may lead to a form of cell damage associated with excessive oxidative injury. This study improves our understanding of how reptiles respond to rising temperatures and provides valuable information for predicting the biological impacts of climate change and supporting future wildlife conservation.

## 1. Introduction

Global warming and climate change are increasing average global temperatures and the frequency of extreme weather events, thereby affecting ecosystems and the survival of living organisms worldwide [1,2]. These changes affect not only environmental composition but also the long-term structure and function of ecosystems [3,4]. In particular, ectothermic animals such as reptiles, which are unable to regulate their own body temperature, are directly influenced by environmental temperature in terms of physiology, behavior, and survival [5].

**Figure 1:**
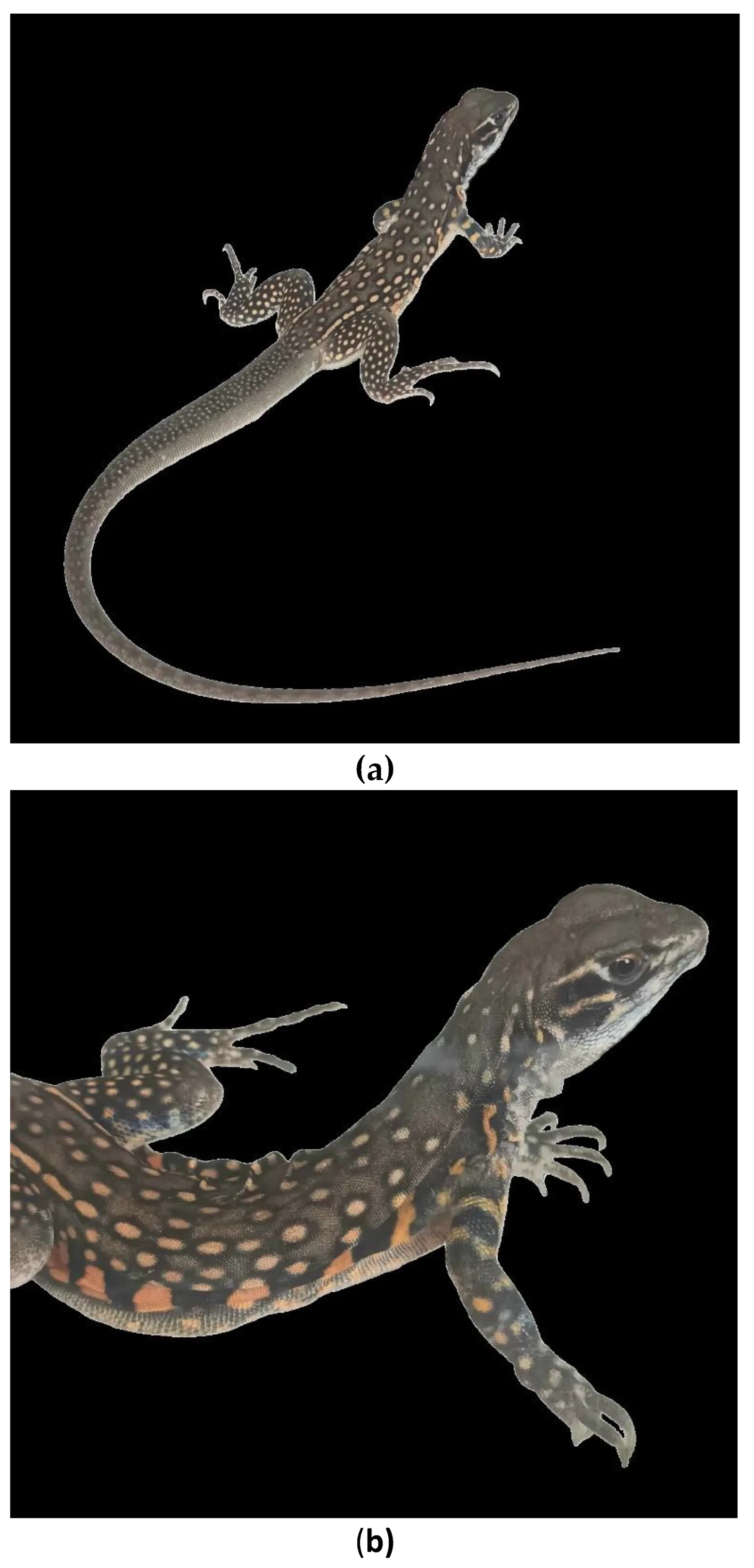
Butterfly lizard, *Leiolepis belliana belliana*: (**a**) whole body; (**b**) lateral view.

*Leiolepis belliana belliana* (Figure 1) is a burrowing lizard of the family Agamidae that inhabits open sandy habitats and open lowland dipterocarp forests. These lizards construct burrows in sandy soil and retreat into them when disturbed [6]. Nevertheless, increasing environmental temperature remains a major environmental challenge for ectothermic animals because elevated temperatures increase metabolic demand, respiratory activity, and energy expenditure [7,8].

At the cellular level, a general mechanism involved in the response to heat stress is altered cellular metabolic activity, which plays a key role in energy production through oxidative phosphorylation. Under high-temperature conditions, increased cellular energy demand may enhance the activity of the mitochondrial electron transport chain (ETC) to generate ATP. However, increased ETC activity is also associated with electron leakage, particularly from complexes I and III, which are major sources of reactive oxygen species [9]. When reactive oxygen species are generated excessively, oxidative stress can occur because of an imbalance between reactive oxygen species production and antioxidant defense systems, leading to damage to cellular components such as lipids, proteins, and DNA [10]. Nitric oxide is another important molecule involved in redox homeostasis, and its level depends on the balance between production and elimination [11]. Nitric oxide can exert both antioxidant and pro-oxidant effects, depending on its intracellular concentration [12].

At the cellular level, iron homeostasis is another general mechanism closely linked to oxidative stress, particularly through the balance between ferrous and ferric iron within cells. Ferrous iron participates in the Fenton reaction, leading to the production of highly toxic hydroxyl radicals [13]. When iron homeostasis becomes disrupted, it promotes reactive oxygen species generation and lipid peroxidation, which may subsequently damage cell membranes and intracellular components. This process is associated with ferroptosis, an iron-dependent form of cell death characterized by the accumulation of lipid peroxidation, in which glutathione and glutathione peroxidase play key roles in its inhibition [14,15]. Although there is currently no direct evidence linking heat stress to ferroptosis in reptiles, available evidence suggests that heat stress may increase reactive oxygen species production through alterations in cellular metabolism. Increased mitochondrial ROS production and lipid peroxidation are, in turn, mechanistically associated with ferroptosis-related processes [16].

Collectively, previous studies indicate that cellular metabolic activity, redox balance, and iron homeostasis are closely interconnected under high-temperature conditions. Mitochondrial dysfunction is a major source of mitochondrial reactive oxygen species, contributing to oxidative stress and lipid peroxidation, whereas iron dysregulation may play an important role in amplifying oxidative damage and lipid peroxidation, processes that are mechanistically associated with ferroptosis [17]. Nitric oxide and iron are key mediators involved in regulating and aggravating oxidative damage under oxidative stress conditions. Excessive nitric oxide can react with superoxide radicals to generate reactive nitrogen species, such as peroxynitrite, resulting in more severe intracellular oxidative damage and redox imbalance [18]. However, in reptiles, particularly *Leiolepis belliana belliana*, information regarding the relationship between physiological changes and these systems under acute thermal elevation, including the potential effects on redox balance, iron metabolism, and cellular damage, remains limited. Such information may help clarify temperature response mechanisms in ectothermic animals and improve our understanding of the impact of climate change on this group of organisms.

Therefore, this study hypothesized that increased environmental temperature would induce physiological changes, enhance cellular metabolic activity, and increase reactive oxygen species production, together with alterations in nitric oxide and iron homeostasis, ultimately leading to redox imbalance and cellular damage. Accordingly, this study aimed to investigate physiological changes, oxidative imbalance, antioxidant systems, nitric oxide, iron metabolism, and red blood cell morphology under elevated temperature conditions in butterfly lizards. Both in vivo and in vitro responses were investigated to elucidate the physiological and cellular effects of acute temperature elevation in this ectothermic species. The findings may improve our understanding of temperature response mechanisms in reptiles, provide insight into the biological impacts of climate change, and serve as a basis for future studies on cellular mechanisms and conservation strategies.

## 2. Materials and Methods

### 2.1. Animals

This study was conducted between February and April 2026. Ten healthy male butterfly lizards (*Leiolepis belliana belliana*), weighing 30–50 g, were used in this study. Their body weights and morphometric measurements, including head length, trunk length, tail length, and chest circumference, are provided in Table S1. Five animals were randomly selected for repeated physiological measurements to evaluate the effects of increasing environmental temperature. The sample size was determined according to the principles of the 3Rs (Replacement, Reduction, and Refinement) to minimize animal use [19] while permitting repeated measurements in the same individuals. Following completion of the physiological experiment, blood samples collected from all 10 butterfly lizards were used for the in vitro biochemical analyses.

Butterfly lizards were purchased from farms in Mahasarakham Province. Species identification was based on external morphology according to Hartmann et al. [6]. The butterfly lizards were transported in nylon bags, which were sealed for ease of handling. Upon arrival at the laboratory, they were placed in prepared containers for temporary rearing. A veterinarian then performed a health examination to confirm that the animals were healthy and showed no abnormalities. After the health check, the butterfly lizards were transferred to a greenhouse. The rearing area consisted of plastic containers measuring 60 cm in diameter and 80 cm in height, with the tops covered with netting to prevent escape while allowing good ventilation. Inside each container, a mixture of soil and sand was added to a depth of approximately 10 cm, together with 30 cm long plastic pipes (5.08 cm diameter) containing holes to provide shelter from light and disturbances. Two butterfly lizards were housed in each plastic container, and trays containing clean water were provided at all times. The butterfly lizards were also kept away from direct sunlight.

The butterfly lizards were reared for 14 days to acclimate to the husbandry and laboratory environment before the experiment began; they were not acclimated to the experimental temperatures during this period. During this period, the butterfly lizards were trained to become familiar with observation procedures, including handling, placement in restraint bags, and movement, in order to minimize stress during the experiment. In addition, the butterfly lizards were gradually accustomed to the actual experimental procedures. Physiological observations were performed according to the same procedures used during the experiment, including respiratory rate, pulse rate, blood oxygen saturation, and body temperature measurements. During the adaptation period, the butterfly lizards were fed insects, larvae, and fresh grass once daily in the morning between 8:00 and 9:00 a.m., at an amount equivalent to approximately 3–5% of their body weight. Any leftover food was removed in the evening.

### 2.2. Experimental Design

This research was divided into two sub-experiments:

#### 2.2.1. Sub-Experiment 1: Effects of Progressive Environmental Temperature Elevation on Physiological Responses in Butterfly Lizards

The physiological experiment was designed as a sequential cumulative thermal elevation protocol to simulate the gradual increase in ambient temperature under natural daytime conditions. The same five butterfly lizards were exposed sequentially to progressively increasing temperatures from 27 to 37 °C, and physiological variables were measured repeatedly at each temperature level. No washout period was applied between temperature levels because the objective was to evaluate cumulative physiological responses to progressive thermal elevation. A repeated-measures experimental design was used with six temperature treatments at 2 °C intervals (27, 29, 31, 33, 35, and 37 °C). At each temperature level, the same five butterfly lizards were evaluated repeatedly. For each treatment, dry glass enclosures were placed in temperature-controlled water baths (Memmert, Schwabach, Germany). Butterfly lizards were individually placed in nylon bags, which were then sealed and transferred to the glass enclosures. The animals were maintained at each experimental temperature for 20 min before physiological measurements were performed. Immediately after the 20 min exposure period, respiratory rate was recorded while the lizard remained in the restraint bag. The lizard was then removed from the bag and gently restrained by hand for measurement of pulse rate, blood oxygen saturation, and body temperature.

#### 2.2.2. Sub-Experiment 2: Effects of Progressive Environmental Temperature Elevation on Biochemical and Red Blood Cell Morphometric Variables in Butterfly Lizards

The biochemical experiment included seven temperature levels at 2 °C intervals (27, 29, 31, 33, 35, 37, and 39 °C). Red blood cell morphometric measurements were performed at six temperatures (27, 29, 31, 33, 35, and 37 °C). For each temperature, biochemical measurements were obtained from five technical/exposure replicates derived from the same pooled blood preparation. The experimental procedure was as follows: Blood was collected by cardiac puncture, a blood-sampling approach previously reported in lizards [20]. The lizards were anesthetized, and cardiac puncture was performed using a 1 mL syringe fitted with a 26-gauge, 1.5-inch needle. Blood was gently aspirated and transferred into heparin-containing blood collection tubes. An intended maximum of 0.5 mL of blood was collected from each animal. Approximately 2.5 mL of usable blood was obtained from the 10 lizards and pooled for the subsequent in vitro experiment. Blood cell preparation was performed according to Jinagool et al. [21]. Briefly, the blood cells were washed twice with PBS (pH 7.4) by centrifugation at 2500 rpm (Hettich Rotina 380R, Hettich GmbH & Co. KG, Tuttlingen, Germany) for 5 min, with the clear supernatant discarded after each centrifugation. The washed blood cells were then diluted with PBS (pH 7.4) at a ratio of 1:200. The diluted blood samples were divided into groups of five test tubes, with 10 mL per tube, to serve as technical replicates for each temperature during biochemical measurements. These replicate tubes were derived from the same pooled blood preparation and therefore represented technical/exposure replicates rather than independent biological replicates. The diluted blood samples were then placed in a temperature-controlled water bath (Memmert, Schwabach, Germany) for progressive thermal exposure, following an experimental approach previously used to evaluate temperature-dependent responses in blood cells [21].

The temperature range selected for this study was intended to mimic environmental temperature changes experienced by butterfly lizards in nature under hot weather conditions. The diluted blood samples were first maintained at 27 °C for 30 min for acclimation. The temperature was then increased at 2 °C intervals to 29, 31, 33, 35, 37, and 39 °C, respectively. At each temperature, the diluted blood was maintained for a standardized 20 min exposure period to evaluate acute cellular responses during progressive thermal elevation. This interval was selected as part of the experimental design to provide a consistent exposure duration at each temperature step and was not intended to represent a species-specific physiological threshold. After the specified incubation period at each temperature, five designated tubes were removed for subsequent biochemical analyses, while the remaining tubes continued through the progressive temperature sequence. One mL of diluted blood from each tube was then transferred into 1.5 mL tubes for cellular metabolic activity analysis. The remaining blood was centrifuged to obtain the supernatant, which was transferred into 1.5 mL tubes and stored at approximately −20 °C. The supernatant was then analyzed for biochemical parameters, including nitric oxide, hydrogen peroxide, malondialdehyde, glutathione, vitamin E, free iron (Fe^2+^/Fe^3+^), total antioxidant capacity, catalase activity, glutathione peroxidase activity, and protein oxidation. For red blood cell morphometric analysis, three sedimented blood cell samples were randomly selected at each temperature from 27 to 37 °C (27, 29, 31, 33, 35, and 37 °C). Red blood cell morphology was not evaluated at 39 °C.

### 2.3. Determination of Indicators

#### 2.3.1. Physiological Parameters

##### Respiratory Rate

Respiratory rate was measured by observing the movement of the anterior rib cage. One complete respiratory cycle was counted as one respiration. The respiratory rate was recorded over a 1 min observation period [22].

##### Pulse Rate and Blood Oxygen Saturation

Pulse rate and blood oxygen saturation were measured using a pulse oximeter (VE-H100B, Edan, Shenzhen, China). The sensor was placed on the webbing between the toes of the left hind leg of the butterfly lizard.

##### Body Temperature

Body temperature was measured using a JENWAY 3345 Ion Meter probe (Jenway, Staffordshire, UK). The probe was inserted into the cloacal opening of the butterfly lizard to a depth of approximately 1 cm for 1 min, after which the body temperature was recorded.

#### 2.3.2. Biochemical Parameters

##### MTT-Reducing Activity

Cellular metabolic activity was measured as follows: 5 mg/mL MTT Formazan (Sigma-Aldrich, Saint Louis, MO, USA) was dissolved and filtered before use. One mL of diluted blood was placed in a 1.5 mL sample tube and centrifuged at 4 °C at 2500 rpm. Then, 900 µL of the supernatant was aspirated. The blood cells were transferred into a microplate (Corning 96-well flat-bottom microplate, Corning, NY, USA), and 10 µL of MTT solution was added to each well. The samples were incubated at 25 °C for 75 min. After incubation, the supernatant was aspirated, and dimethyl sulfoxide (Loba Chemie Pvt. Ltd., Mumbai, India) was added. Absorbance was measured at 540 nm using a Tecan Infinite^®^ 200 Microplate Reader (Tecan Trading AG, Männedorf, Switzerland) [23].

##### Hydrogen Peroxide

Hydrogen peroxide levels were measured according to the method of Orprayoon et al. [24]. One mL of 2.25 mM FeSO_4_·7H_2_O (Ajax Finechem Pty. Ltd., Taren Point, Australia) was added into a test tube, followed by 1 mL of supernatant. The mixture was mixed thoroughly and incubated for 5 min. Then, 1 mL of 4 mM norfloxacin (Sigma-Aldrich, Saint Louis, MO, USA) was added, mixed again, and incubated for another 3 min. Absorbance was measured at 440 nm using a Tecan Infinite^®^ 200 Microplate Reader (Tecan Trading AG, Männedorf, Switzerland), with hydrogen peroxide (QRëC™, Auckland, New Zealand) used as the standard.

##### Nitric Oxide

Nitric oxide levels were measured using the Griess reagent method. The Griess solution consisted of 1% sulfanilamide (DC Fine Chemicals, Terrassa, Spain), 0.1% N-(1-naphthyl) ethylenediamine dihydrochloride (PanReac AppliChem, Darmstadt, Germany), and 1.25% phosphoric acid (QRëC™, Auckland, New Zealand). Distilled water was added to obtain a final volume of 100 mL, and the solution was shaken well. Equal volumes of the Griess solution and supernatant were mixed and incubated at room temperature for 15 min. Absorbance was measured at 540 nm using a Tecan Infinite^®^ 200 Microplate Reader (Tecan Trading AG, Männedorf, Switzerland), with sodium nitrite (KemAus, Cherrybrook, Australia) used as the standard [25].

##### Total Antioxidant Capacity

Total antioxidant capacity levels were measured by first preparing a solution consisting of 300 mM sodium acetate buffer (pH 3.6) (Ajax Finechem Pty. Ltd., Taren Point, Australia), 40 mM HCl (QRëC™, Auckland, New Zealand), 10 mM TPTZ (2,4,6-tri [2-pyridyl]-s-triazine) (Sigma-Aldrich, Saint Louis, MO, USA), and 20 mM FeCl_3_·6H_2_O (PanReac AppliChem, Darmstadt, Germany). The FRAP reaction solution was then prepared by mixing 10 mL of 300 mM acetate buffer with 1 mL of 10 mM TPTZ and 1 mL of 20 mM FeCl_3_·6H_2_O. Twenty µL of the supernatant was mixed with 180 µL of freshly prepared FRAP solution in a microplate (Corning 96-well flat-bottom microplate, Corning, NY, USA) and incubated at room temperature for 5 min. Absorbance was measured at 595 nm using a Tecan Infinite^®^ 200 Microplate Reader (Tecan Trading AG, Männedorf, Switzerland), with FeSO_4_·7H_2_O (Ajax Finechem Pty. Ltd., Taren Point, Australia) used as the standard [26].

##### Glutathione

The measurement of glutathione levels was adapted from the method of Ellman [27]. The procedure began with the preparation of a 10 mM 5,5′-dithiobis-(2-nitrobenzoic acid) (DTNB) (Sigma-Aldrich, Saint Louis, MO, USA; Made in Switzerland) stock solution using ethanol as a solvent. A 0.125 mM DTNB working solution was then prepared by mixing 1.25 mL of DTNB stock solution with 98.75 mL of distilled water. For the assay procedure, 235 µL of DTNB working solution and 15 µL of sample were added into the wells of a Corning 96-well flat-bottom microplate (Corning, NY, USA), resulting in a total volume of 250 µL per well. The mixture was incubated at room temperature for 5 min, after which absorbance was measured at 412 nm using a Tecan Infinite^®^ 200 Microplate Reader (Tecan Trading AG, Männedorf, Switzerland). L-glutathione reduced (Sigma-Aldrich, Shanghai, China) was used as the standard substance.

##### Glutathione Peroxidase Activity

The measurement of glutathione peroxidase activity was adapted from the method of Rotruck et al. [28]. The procedure began with the preparation of 50 mM phosphate buffer (pH 7.0), together with 1 mM L-glutathione reduced (3.073 mg/10 mL) (Sigma-Aldrich, Shanghai, China), 2.5 mM hydrogen peroxide (QRëC™, Auckland, New Zealand), and 1 mM 5,5′-dithiobis-(2-nitrobenzoic acid) (DTNB) (Sigma-Aldrich, Saint Louis, MO, USA; Made in Switzerland) stock solutions prepared in the same buffer. For the GSH control, 150 µL phosphate buffer, 30 µL L-glutathione reduced, 20 µL hydrogen peroxide, and 20 µL sample were mixed and incubated at 37 °C for 5–10 min. Then, 30 µL DTNB was added, and absorbance was measured at 412 nm using a Tecan Infinite^®^ 200 Microplate Reader (Tecan Trading AG, Männedorf, Switzerland). For the sample assay, the sample was used instead of phosphate buffer. The amount of GSH used was calculated using the following equation:

GSH used = GSH control − GSH remaining

Protein concentration was quantified using the Bradford assay. The Bradford reagent was prepared by dissolving 10 mg of Coomassie Brilliant Blue G-250 (PanReac AppliChem, Darmstadt, Germany) in 5 mL of ethanol (Merck, Darmstadt, Germany), followed by the addition of 10 mL of phosphoric acid (QRëC™, Auckland, New Zealand) and distilled water to obtain a final volume of 100 mL before filtration. For the assay, 20 µL of sample was mixed with 200 µL of Bradford reagent and incubated at room temperature for 5–10 min. Absorbance was measured at 595 nm using a Tecan Infinite^®^ 200 Microplate Reader (Tecan Trading AG, Männedorf, Switzerland). Bovine serum albumin (Sigma-Aldrich, Saint Louis, MO, USA) was used as the standard. Glutathione peroxidase activity was then expressed as U/mg protein using the following equation:

GPx activity (U/mg protein) = GSH used (nmol)/[Incubation time (min) × Protein (mg)]

##### Catalase Activity

The measurement of catalase activity was adapted from the method of Goth [29]. The procedure began with the preparation of 50 mM phosphate buffer at pH 7.0 for pH adjustment and sample dilution. A 20 mM hydrogen peroxide solution was prepared from 35% hydrogen peroxide (QRëC™, Auckland, New Zealand), and a dichromate/acetic acid reagent was prepared by mixing 5% potassium dichromate solution (UNILAB^®^, Sydney, Australia) with acetic acid (QRëC™, Auckland, New Zealand) at a ratio of 1:1 (*v*/*v*). The assay was performed by adding 1000 µL of hydrogen peroxide solution and 50 µL of sample into a test tube and incubating at 37 °C for 60 s before stopping the reaction. Then, 1000 µL of dichromate/acetic acid reagent was added, and the mixture was boiled in water for 10 min. Absorbance was measured at 570 nm using a Tecan Infinite^®^ 200 Microplate Reader (Tecan Trading AG, Männedorf, Switzerland), with hydrogen peroxide used as the standard.

##### Vitamin E

The measurement of vitamin E was adapted from the method of Martinek [30], beginning with the preparation of 0.12% (*w*/*v*) TPTZ (2,4,6-tri [2-pyridyl]-s-triazine) (Sigma-Aldrich, Saint Louis, MO, USA) and 0.12% (*w*/*v*) FeCl_3_ (PanReac AppliChem, Darmstadt, Germany) solutions prepared in n-propanol (KemAus™, Cherrybrook, Australia) and ethanol (Merck, Darmstadt, Germany), respectively. Four hundred µL of each sample was mixed with 400 µL each of ethanol and xylene (Labscan Analytical Science, Dublin, Ireland), shaken vigorously for 30 s, and centrifuged at 600 RCF for 10 min. A 200 µL aliquot of the upper layer was collected for the reaction and mixed with 200 µL of TPTZ solution. After 4 min, 40 µL of FeCl_3_ solution was added. The mixture was shaken and allowed to stand for 12 min before 200 µL was transferred into a microplate (Corning 96-well flat-bottom microplate, Corning, NY, USA) for absorbance measurement at 600 nm using a Tecan Infinite^®^ 200 Microplate Reader (Tecan Trading AG, Männedorf, Switzerland). D-α-tocopherol succinate (Glentham Life Sciences, Corsham, UK) was used as the standard.

##### Malondialdehyde

To measure malondialdehyde, 0.01 mL of sample was mixed with 3 mL of 0.05 mol/L HCl (QRëC™, Auckland, New Zealand) and 1 mL of 0.67% thiobarbituric acid (Merck, Beijing, China). The mixture was heated at 100 °C for 30 min and then cooled under running water. After cooling, 4 mL of n-butyl alcohol (QRëC™, Auckland, New Zealand) was added, and the mixture was shaken thoroughly using a vortex mixer (Vortex Genie 2, Scientific Industries, Bohemia, NY, USA). The samples were then centrifuged at 3000 rpm (1008× *g*) using a Hettich Rotina 380R centrifuge (Hettich GmbH & Co. KG, Tuttlingen, Germany) for 10 min. Absorbance was measured at 532 nm using a Tecan Infinite^®^ 200 Microplate Reader (Tecan Trading AG, Männedorf, Switzerland), with 1,1,3,3-tetramethoxypropane (Merck, Beijing, China) used as the standard [31].

##### Protein Oxidation

Protein oxidation was measured using a modified method from Witko-Sarsat et al. [32]. A 1.16 M potassium iodide solution (AnalaR, Leicestershire, UK) was prepared. Two hundred µL of sample was added to a microplate (Corning 96-well flat-bottom microplate, Corning, NY, USA), followed by 20 µL of acetic acid, 10 µL of 1.16 M potassium iodide, and an additional 20 µL of acetic acid (QRëC™, Auckland, New Zealand). The mixture was thoroughly mixed and incubated at room temperature. Absorbance was then measured at 340 nm using a Tecan Infinite^®^ 200 Microplate Reader (Tecan Trading AG, Männedorf, Switzerland), and chloramine-T (Loba Chemie Pvt. Ltd., Mumbai, India) was used as the standard.

##### Ferrous Ion

The measurement of ferrous iron was adapted from the method of Fortune and Mellon [33]. A 1000 ppm o-phenanthroline solution (QRëC™, Auckland, New Zealand) was prepared. Acetate buffer at pH 4.5 was prepared by dissolving 2.72 g of sodium acetate trihydrate (QRëC™, Auckland, New Zealand) in 80 mL of distilled water, and the pH was adjusted using acetic acid (QRëC™, Auckland, New Zealand). Distilled water was then added to obtain a final volume of 100 mL. The assay procedure was performed by mixing 300 µL of o-phenanthroline, 300 µL of acetate buffer, 1000 µL of acetone (Merck, Darmstadt, Germany), 300 µL of distilled water, and 100 µL of sample. The mixture was shaken and allowed to stand until color development occurred. Then, 100 µL of the solution was transferred into a microplate (Corning 96-well flat-bottom microplate, Corning, NY, USA), and absorbance was measured at 510 nm using a Tecan Infinite^®^ 200 Microplate Reader (Tecan Trading AG, Männedorf, Switzerland). FeSO_4_·7H_2_O (Ajax Finechem Pty. Ltd., Taren Point, Australia) was used as the standard.

##### Ferric Ion

The measurement of ferric iron was adapted from the method of Fortune and Mellon [33]. A 100 ppm Na_2_S_2_O_3_ solution (Daejung, Seoul, Republic of Korea) and a 1000 ppm o-phenanthroline solution (QRëC™, Auckland, New Zealand) were prepared. Acetate buffer at pH 4.5 was prepared by dissolving 2.72 g of sodium acetate trihydrate (QRëC™, Auckland, New Zealand). Distilled water was then added to obtain a final volume of 100 mL. The assay procedure was performed by combining 200 µL of Na_2_S_2_O_3_, 300 µL of o-phenanthroline, 300 µL of acetate buffer, 1000 µL of acetone (Merck, Darmstadt, Germany), 100 µL of distilled water, and 100 µL of sample. The mixture was shaken thoroughly. Then, 100 µL of the solution was transferred into a microplate (Corning 96-well flat-bottom microplate, Corning, NY, USA), and absorbance was measured at 510 nm using a Tecan Infinite^®^ 200 Microplate Reader (Tecan Trading AG, Männedorf, Switzerland). Fe_2_(SO_4_)_3_·9H_2_O (KemAus™, Cherrybrook, Australia) was used as the standard.

#### 2.3.3. Morphometry of Red Blood Cells

**Figure 2:**
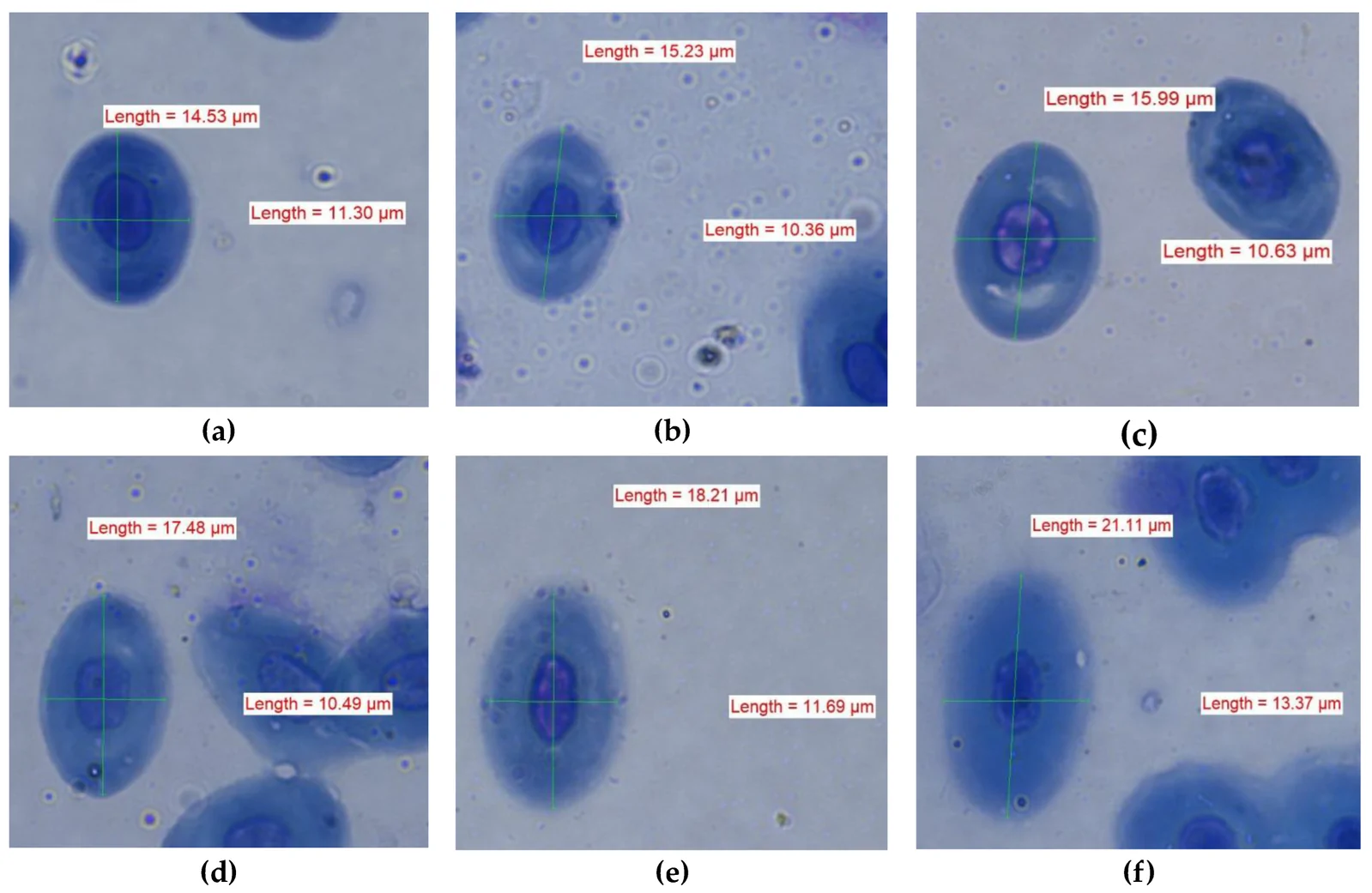
Measurement of the length and width of red blood cells in butterfly lizards exposed to temperatures of 27 °C (**a**), 29 °C (**b**), 31 °C (**c**), 33 °C (**d**), 35 °C (**e**), and 37 °C (**f**).

**Figure 3:**
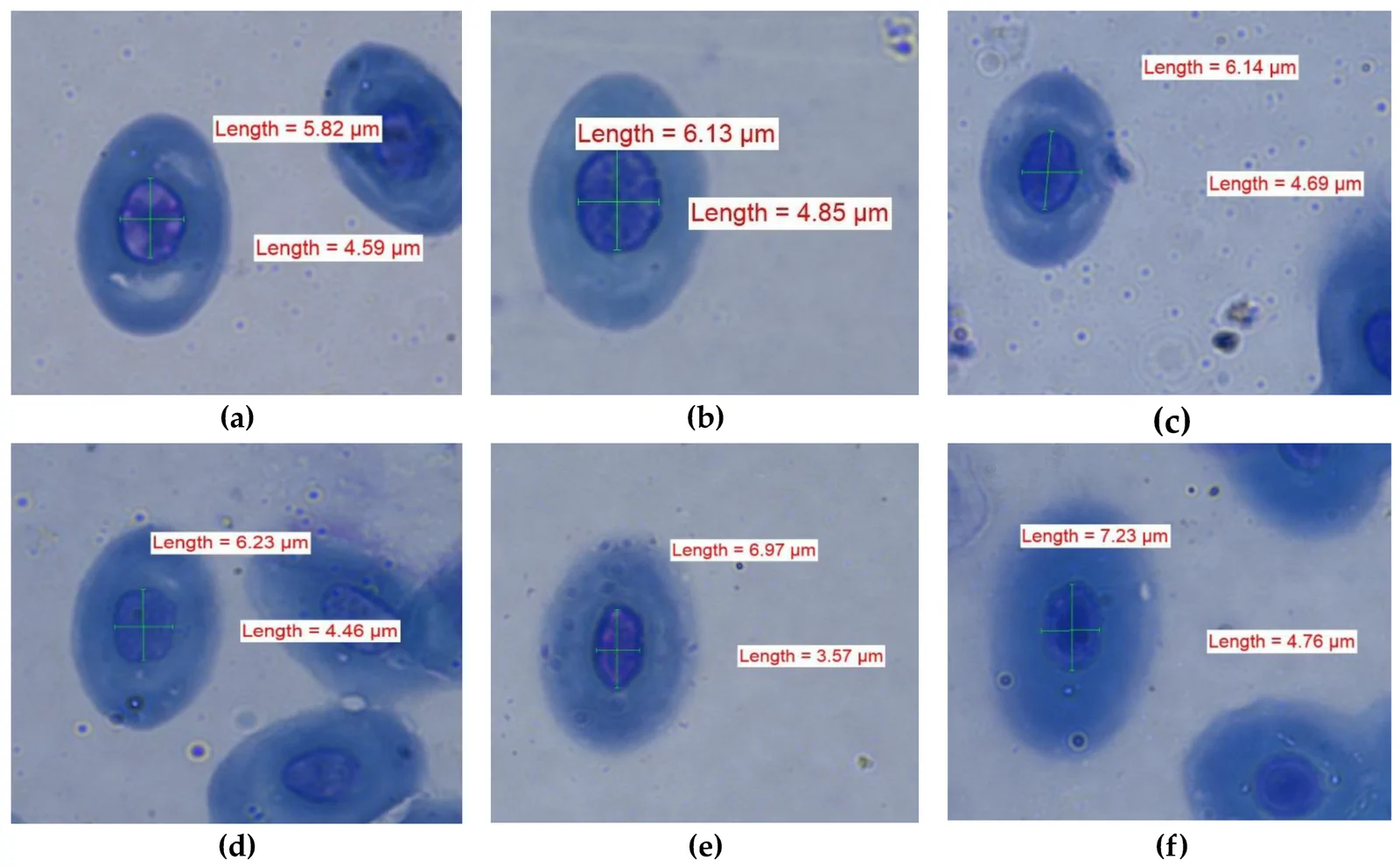
Measurement of the length and width of the nuclei within red blood cells of butterfly lizards exposed to temperatures of 27 °C (**a**), 29 °C (**b**), 31 °C (**c**), 33 °C (**d**), 35 °C (**e**), and 37 °C (**f**).

Red blood cell sediment was smeared on a slide, fixed with methanol, and stained with Giemsa–Wright stain. Cell length (CL) and cell width (CW) (Figure 2), as well as nucleus length (NL) and nucleus width (NW) (Figure 3), were measured using a microscope (Nikon Digital Sight 100 + NIS-Elements software version 5.22.00, Nikon, Tokyo, Japan). Fifty cells were measured per slide. The ratios of cell length to cell width (CL/CW ratio), nucleus length to nucleus width (NL/NW ratio), cell length to nucleus length (CL/NL ratio), and cell width to nucleus width (CW/NW ratio) were calculated.

### 2.4. Statistical Analysis

Data were tested for normality before statistical analysis. Physiological parameters were analyzed using a mixed-effects model, with temperature as the fixed effect and individual lizard as the random effect to account for repeated measurements obtained from the same animals. Biochemical parameters, red blood cell morphometric variables, and morphometric ratios were analyzed using one-way analysis of variance (ANOVA). For the in vitro biochemical data, the replicate tubes represented technical/exposure replicates derived from the same pooled blood preparation; therefore, statistical inference was restricted to temperature-dependent differences within this pooled experimental preparation rather than to biological variation among individual lizards. Each outcome variable was analyzed independently. Pairwise comparisons among temperature groups were performed using Sidak’s multiple comparisons test. Statistical significance was defined at *p* < 0.05. Data are presented as mean ± standard deviation (mean ± SD).

## 3. Results

### 3.1. Body Temperature

**Figure 4:**
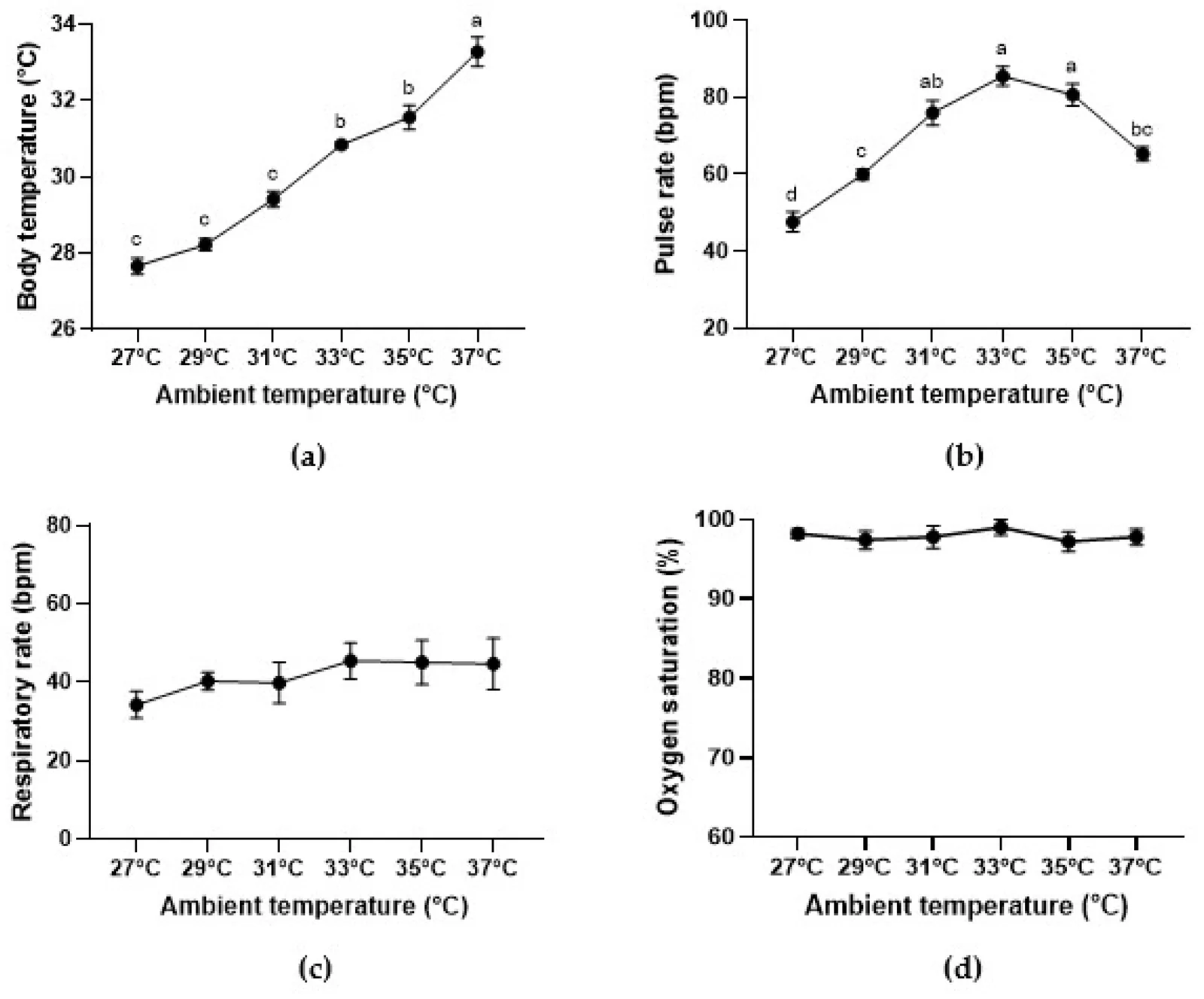
Effects of ambient temperatures ranging from 27 to 37 °C on body temperature (**a**), pulse rate (**b**), respiratory rate (**c**) and oxygen saturation (**d**) of butterfly lizards. Different letters indicate significant differences among temperatures (*p* < 0.05). Data are presented as mean ± SD.

The body temperature of butterfly lizards increased continuously with ambient temperature. At 37 °C, body temperature was significantly higher than that in the 27–35 °C range (*p* < 0.05), while values at 33–35 °C were significantly higher than those at 27–31 °C (*p* < 0.05). No significant difference in body temperature was found between 33 °C and 35 °C, or among temperatures within the 27–31 °C range (*p* > 0.05) (Figure 4a).

### 3.2. Pulse Rate

The pulse rate of butterfly lizards tended to increase with ambient temperature before decreasing slightly at the highest temperature. Pulse rates at 33–35 °C were significantly higher than those at 27–29 °C and 37 °C (*p* < 0.05). At 31 °C, the pulse rate was significantly higher than at 27–29 °C (*p* < 0.05), and values at 29 °C were significantly higher than those at 27 °C (*p* < 0.05). No significant difference in pulse rate was found between 31 and 35 °C or between 31 °C and 37 °C (*p* > 0.05) (Figure 4b).

### 3.3. Respiratory Rate

The respiratory rate of butterfly lizards at temperatures between 27 °C and 37 °C did not differ significantly (*p* > 0.05) (Figure 4c).

### 3.4. Oxygen Saturation

The oxygen saturation of butterfly lizards at temperatures between 27 °C and 37 °C did not differ significantly (*p* > 0.05) (Figure 4d).

### 3.5. MTT-Reducing Activity

**Figure 5:**
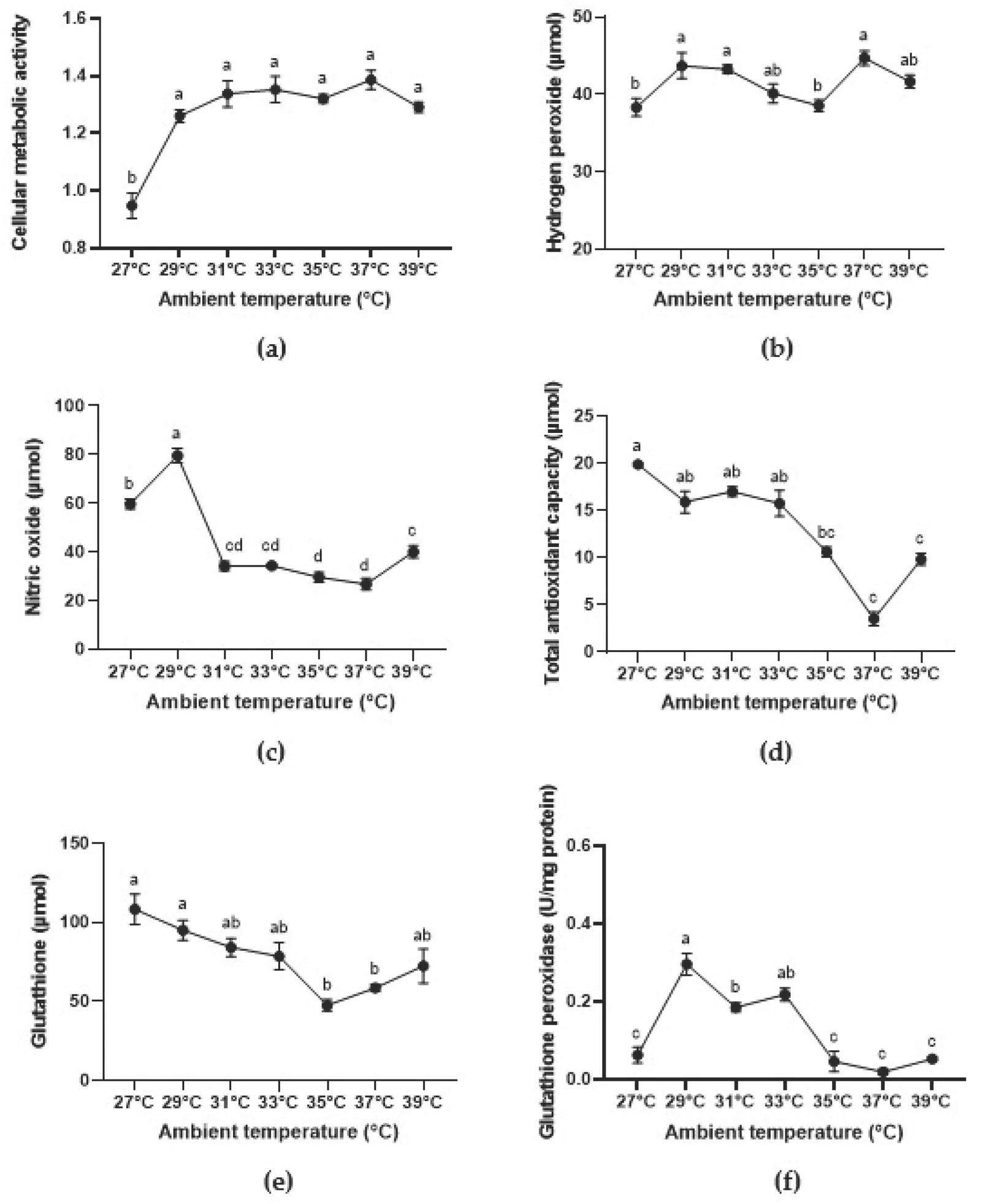
Effects of ambient temperatures ranging from 27 to 39 °C on cellular metabolic activity (**a**), hydrogen peroxide (**b**), nitric oxide (**c**), total antioxidant capacity (**d**), glutathione (**e**) and glutathione peroxidase activity (**f**) of butterfly lizard blood cells. Different letters indicate significant differences among temperatures (*p* < 0.05). Data are presented as mean ± SD.

Cellular metabolic activity in butterfly lizard blood cells increased with temperature and remained relatively constant up to the maximum temperature. Higher cellular metabolic activity was observed at 29–39 °C compared to 27 °C (*p* < 0.05). Differences in cellular metabolic activity among 29–39 °C were not statistically significant (*p* > 0.05) (Figure 5a).

### 3.6. Hydrogen Peroxide

Hydrogen peroxide levels in the blood cells of butterfly lizards changed with increasing ambient temperature. Higher levels (*p* < 0.05) were detected at 29–31 °C and 37 °C compared to 27 °C and 35 °C. No statistically significant difference (*p* > 0.05) was found between 29 and 33 °C and 37 and 39 °C, or between 27 °C and 35 °C (Figure 5b).

### 3.7. Nitric Oxide

Nitric oxide levels in butterfly lizard blood cells varied with ambient temperature. Significantly higher levels were observed at 29 °C compared to 27 °C and the 31–39 °C range (*p* < 0.05). Nitric oxide levels at 27 °C were also significantly higher than those in the 31–39 °C range (*p* < 0.05), while levels at 39 °C were significantly higher than those at 35–37 °C (*p* < 0.05). No statistically significant difference in nitric oxide levels was found within the 31–37 °C range (*p* > 0.05) (Figure 5c).

### 3.8. Total Antioxidant Capacity

Total antioxidant capacity in butterfly lizard blood cells decreased with increasing ambient temperature. Total antioxidant capacity at 27 °C was significantly higher than that at 35–39 °C (*p* < 0.05). Values at 29–33 °C did not differ significantly from either 27 °C or 35 °C (*p* > 0.05), while the value at 35 °C did not differ significantly from those at 29–33 °C or 37–39 °C (*p* > 0.05) (Figure 5d).

### 3.9. Glutathione

Glutathione levels in butterfly lizard blood cells varied with temperature. Levels at 27–29 °C were significantly higher than those at 35–37 °C (*p* < 0.05). The values at 31 °C, 33 °C, and 39 °C did not differ significantly from either the higher or lower groups (*p* > 0.05) (Figure 5e).

### 3.10. Glutathione Peroxidase Activity

Glutathione peroxidase activity in the blood cells of butterfly lizards varied with temperature. Activity at 29 °C was significantly higher than that at 27 °C, 31 °C, and 35–39 °C (*p* < 0.05), but did not differ significantly from that at 33 °C (*p* > 0.05). Activity at 31–33 °C was significantly higher than that at 27 °C and 35–39 °C (*p* < 0.05). No significant differences were observed among 27 °C and 35–39 °C (*p* > 0.05) (Figure 5f).

### 3.11. Catalase Activity

**Figure 6:**
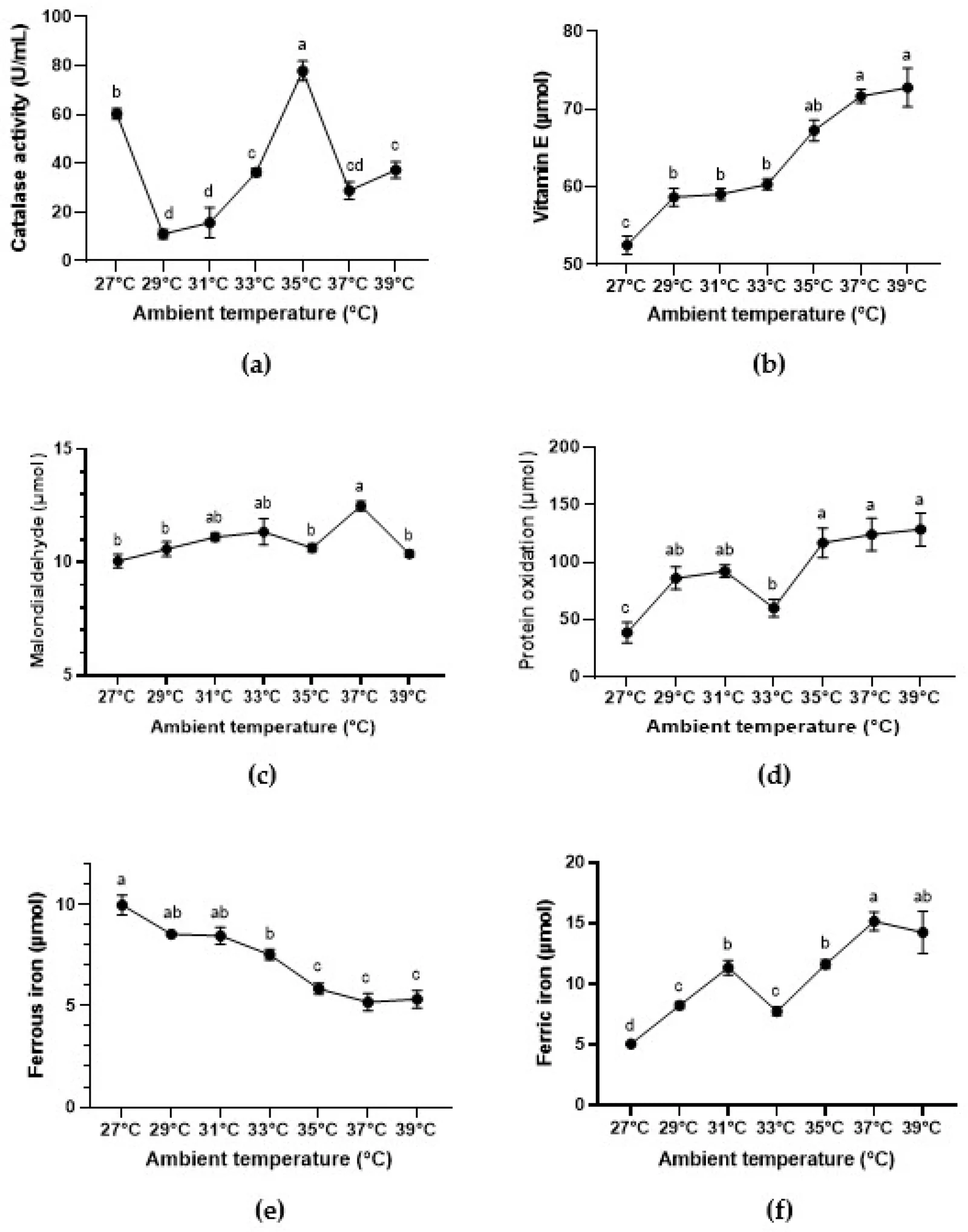
Effects of ambient temperatures ranging from 27 to 39 °C on catalase activity (**a**), vitamin E (**b**), malondialdehyde (**c**), protein oxidation (**d**), ferrous iron (**e**), and ferric iron (**f**) of butterfly lizard blood cells. Different letters indicate significant differences among temperatures (*p* < 0.05). Data are presented as mean ± SD.

Catalase activity in the blood cells of butterfly lizards varied significantly with temperature. Activity was highest at 35 °C and was significantly higher than at all other temperatures (*p* < 0.05). Activity at 27 °C was significantly higher than at 29–33 °C and 37–39 °C (*p* < 0.05). Activity at 33 °C and 39 °C was significantly higher than at 29–31 °C (*p* < 0.05). Catalase activity at 37 °C did not differ significantly from that at 29–33 °C or 39 °C (*p* > 0.05) (Figure 6a).

### 3.12. Vitamin E

Vitamin E levels in butterfly lizard blood cells increased with increasing ambient temperature. Levels at 37–39 °C were significantly higher than those at 27–33 °C (*p* < 0.05), while the level at 35 °C was significantly higher than that at 27 °C (*p* < 0.05). No significant differences were observed among 29–35 °C or among 35–39 °C (*p* > 0.05) (Figure 6b).

### 3.13. Malondialdehyde

Malondialdehyde levels in butterfly lizard blood cells changed with increasing ambient temperature. Significantly higher malondialdehyde levels were found at 37 °C compared to 27–29 °C, 35 °C, and 39 °C (*p* < 0.05). No statistically significant difference (*p* > 0.05) was found between malondialdehyde levels at 31–33 °C and 37 °C, as well as within the 27–35 °C and 39 °C temperature ranges (Figure 6c).

### 3.14. Protein Oxidation

Protein oxidation levels in butterfly lizard blood cells tended to increase with temperature. Levels at 29–39 °C were significantly higher than those at 27 °C (*p* < 0.05). Protein oxidation at 35–39 °C was also significantly higher than that at 33 °C (*p* < 0.05), whereas values at 29–31 °C did not differ significantly from those at either 33 °C or 35–39 °C (*p* > 0.05) (Figure 6d).

### 3.15. Ferrous Iron

Ferrous iron in butterfly lizard blood cells decreased with increasing temperature. The concentration at 27 °C was significantly higher than those at 33–39 °C (*p* < 0.05), while concentrations at 29–31 °C were significantly higher than those at 35–39 °C (*p* < 0.05). The concentration at 33 °C was also significantly higher than those at 35–39 °C (*p* < 0.05). No significant differences were observed among 27–31 °C or among 35–39 °C (*p* > 0.05) (Figure 6e).

### 3.16. Ferric Iron

Ferric iron levels in butterfly lizard blood cells tended to increase with temperature. Significantly higher levels (*p* < 0.05) were observed at 37 °C compared to 27–35 °C, while levels at 39 °C were significantly higher than those at 27–33 °C (*p* < 0.05). No significant differences in ferric iron levels were found between 37 and 39 °C, 31 °C and 35 °C, and between 29 °C and 33 °C (*p* > 0.05) (Figure 6f).

### 3.17. Morphometry

Red blood cell length in butterfly lizards increased with temperature. The highest value was observed at 37 °C. Red blood cell length at 37 °C was significantly greater than that at 27–33 °C (*p* < 0.05), but did not differ significantly from that at 35 °C (*p* > 0.05). The value at 35 °C was significantly greater than those at 27–29 °C (*p* < 0.05), while intermediate temperatures showed overlapping statistical groups. Red blood cell width (CW), red blood cell nucleus length (NL), red blood cell nucleus width (NW), red blood cell length-to-width ratio (CL/CW), nucleus length-to-width ratio (NL/NW), cell length-to-nucleus length ratio (CL/NL), and cell width-to-nucleus width ratio (CW/NW) did not differ significantly among temperatures from 27 to 37 °C (*p* > 0.05) (Table 1).

## 4. Discussion

The body temperature of butterfly lizards increased with increasing ambient temperature, consistent with the characteristic of ectothermic animals in which body temperature is directly influenced by the external environment. The body temperature of these animals is not solely dependent on air temperature, but also on heat energy exchange through solar radiation, convection, conduction, and radiation between the body and the environment [34,35]. Comparable findings have been reported in several lizard species showing that thermal environment or operative temperature is related to the regulation and changes in body temperature, including *Timon lepidus*, *Eremias roborowskii*, *Phrynocephalus axillaris*, *Psammodromus algirus*, and *Sauromalus varius* [34,36,37,38].

The pulse rate of butterfly lizards increased with ambient temperature and peaked at 33–35 °C before decreasing at higher temperatures. This represents a nonlinear response to temperature. This pattern is consistent with a temperature-dependent cardiovascular response and may be associated with increased metabolic and oxygen demands as body temperature rises [38]. However, metabolic rate, oxygen consumption, and cardiac output were not directly measured in the present study; therefore, this interpretation should be considered a possible physiological explanation rather than a directly demonstrated mechanism. In ectotherm animals, the relationship between pulse rate and oxygen consumption may not always be linear, as pulse rate can change in response to thermoregulation and changes in body temperature even without a proportional increase in metabolic rate [39,40]. The decrease in pulse rate at 37 °C may reflect entry into the temperature range where thermal limitation begins, consistent with the concept of the thermal performance curve, which states that physiological efficiency increases until an optimal temperature range is reached before declining when temperature exceeds physiological limits [41]. The observed decrease in pulse rate at 37 °C may also be related to an adjustment in cardiovascular function during exposure to elevated temperature [42]. This interpretation is consistent with the observed pulse-rate pattern, which peaked at 33–35 °C and subsequently decreased at 37 °C. The pulse rates observed in the present study represent temperature-specific experimental responses and should not be interpreted as a normal reference range for *Leiolepis belliana belliana*, as a species-specific physiological reference interval has not yet been established.

The respiration rate of butterfly lizards within the temperature range of 27–37 °C was not statistically different, although the mean tended to increase with increasing temperature, but this change was not as pronounced as the pulse rate. This may indicate that the respiratory system can still maintain gas exchange equilibrium within the studied temperature range without necessarily increasing respiratory frequency. In the tegu lizard (*Tupinambis merianae*), the cardiorespiratory response to temperature is not determined by changes in any single variable alone, but rather reflects the combined effects of body temperature, pulse rate, respiration, and oxygen consumption [7]. As temperature increases, limitations in oxygen supply and oxygen demand may begin to influence the physiological performance of ectotherm animals [40]. In this study, no significant increase in respiratory rate was found, suggesting that temperatures of 27–37 °C do not yet elicit a pronounced ventilatory response in butterfly lizards.

The blood oxygen saturation of butterfly lizards did not differ significantly under the studied temperatures, which may reflect that the oxygen transport and utilization systems can maintain equilibrium within the studied temperature range, despite slight fluctuations at each temperature. In general, tissue oxygenation results from a balance between tissue oxygen delivery and oxygen consumption, rather than being a constant value [42]. At elevated temperatures, the circulatory system and oxygen transport may adapt to respond to increased tissue oxygen demand [43,44]. Blood oxygen saturation is influenced by the balance among circulatory blood flow, oxygen transport, and tissue oxygen utilization [45,46]. Therefore, the fact that blood oxygen saturation values did not change significantly may suggest that the physiology of butterfly lizards can still maintain a balance between oxygen transport and oxygen utilization within the experimental temperature range.

Cellular metabolic activity in butterfly lizards increased with increasing temperature, with cellular metabolic activity at 29–39 °C being higher than at 27 °C. These findings suggest a response of the intracellular energy system to increasing environmental temperature, as temperature can affect mitochondrial processes, mitochondrial respiration, and metabolic activity in ectotherm animals [47]. The increase in cellular metabolic activity may be related to increased production of reactive oxygen species and alterations in intracellular oxidative balance [48,49]. This interpretation is also supported by the observed temperature-dependent changes in hydrogen peroxide levels in the present study. Alterations in cellular metabolism and the accumulation of reactive oxygen species are also associated with lipid peroxidation, a process that is mechanistically linked to ferroptosis in several biological systems [50,51].

Hydrogen peroxide levels also changed with temperature. Hydrogen peroxide levels increased at certain temperatures, decreased at 35 °C, and increased again at 37 °C. Nitric oxide levels increased during the initial temperature range before decreasing as temperature increased. This phenomenon may reflect that, as ambient temperature increases, oxidative imbalance and reactive oxygen species accumulation may occur in the blood cells of butterfly lizards. Hydrogen peroxide is a reactive oxygen species produced from the conversion of superoxide radicals through antioxidant defense and can be converted into hydroxyl radicals via the Fenton reaction [10,51]. Nitric oxide can also react with reactive oxygen species to produce reactive nitrogen species, which are involved in changes in cellular redox regulation [10].

Ferrous iron levels decreased while ferric iron increased as ambient temperature increased. This may indicate a shift in the iron redox state towards a more oxidized form, where ferrous iron can react with hydrogen peroxide via the Fenton reaction to generate hydroxyl radicals before being oxidized to ferric iron [52,53,54]. Therefore, the decrease in ferrous iron, together with the increase in ferric iron and hydrogen peroxide, may be related to iron redox cycling and reactive oxygen species-mediated oxidative processes during exposure to elevated environmental temperatures.

As for nitric oxide, nitric oxide initially increased and then decreased as ambient temperature continued to rise. This may occur because nitric oxide can react with reactive oxygen species, particularly superoxide, to produce reactive nitrogen species such as peroxynitrite, resulting in decreased nitric oxide bioavailability [55]. The nitric oxide response to heat stress may also vary with thermal conditions and tissue-specific physiological responses [56], and such changes may reflect a balance between nitric oxide production and utilization within the cell rather than any single factor alone [57]. Comparable findings were reported by Jinagool et al. [21] in tilapia (*Oreochromis niloticus* Linn.), where nitric oxide levels decreased in the temperature range of 28–32 °C before increasing and decreasing again as temperature continued to rise, demonstrating that nitric oxide can change according to the cellular response under different temperature conditions.

The total antioxidant capacity of butterfly lizards decreased with increasing ambient temperature. The total antioxidant capacity at 27 °C was higher than at 35–37 °C, indicating that the intracellular antioxidant system begins to lose reducing capacity under high-temperature environmental conditions. Generally, total antioxidant capacity is an indicator of the oxidant-buffering capacity and reducing power of the intracellular antioxidant system, reflecting the ability to maintain oxidative balance and mitigate the effects of intracellular oxidants [58]. The decrease in total antioxidant capacity at elevated temperatures may be related to reactive oxygen species accumulation during thermal stress, leading to increased utilization of the antioxidant defense system and decreased cellular reducing capacity [59]. This finding differs from the report by Zhang et al. [5] in the desert lizard *Phrynocephalus przewalskii*, which found that heatwaves can induce oxidative stress and oxidative damage even though total antioxidant capacity did not change significantly. A study by Valgas et al. [60] on *Liolaemus arambarensis* also found that heat stress could activate antioxidant enzymes, although possibly not sufficiently to prevent oxidative damage. These differences among studies may reflect species-specific thermal physiology and adaptation, as well as differences in baseline thermal conditions, the magnitude and duration of heat exposure, and experimental design. These factors may influence the capacity and timing of antioxidant responses during thermal stress.

Glutathione tended to decrease with increasing temperature, reaching a minimum at 35 °C. Glutathione peroxidase activity increased in the initial to intermediate temperature range, most notably at 29–33 °C, before declining at elevated temperatures. These findings suggest that the butterfly lizard GSH–GPx system may initially respond to oxidative challenges but gradually loses efficiency as temperature continues to increase. Generally, glutathione peroxidase uses glutathione as a reducing substrate to reduce hydrogen peroxide and lipid hydroperoxides to less toxic forms [50]. The GSH–GPx system plays a crucial role in regulating reactive oxygen species, limiting lipid peroxidation, and helping maintain intracellular oxidative balance [48,50,61]. The decrease in glutathione during oxidative stress may therefore reflect the utilization of glutathione to support the function of glutathione-dependent antioxidant enzymes, reducing the ability to scavenge peroxides and lipid hydroperoxides, and may be associated with increased accumulation of oxidative intermediates and lipid peroxidation [50]. Comparable findings were reported by He et al. [62] in the high-altitude frog *Nanorana pleskei*, which found that under high temperatures, the activity of glutathione peroxidase and several antioxidant enzymes increased, whereas under heat-wave conditions, the activity of most antioxidant enzymes decreased. This may indicate that the antioxidant system of ectotherm animals can respond to high temperatures to some extent, but such responses may decline when exposed to more severe or fluctuating heat.

Catalase enzyme activity in butterfly lizards was also temperature-dependent. Catalase activity initially decreased before peaking at 35 °C, which may correlate with the decreased hydrogen peroxide levels within this temperature range. Catalase is a crucial enzyme that converts hydrogen peroxide into water and oxygen to reduce oxidative damage within cells [63,64]. Antioxidant responses to increasing temperature may vary depending on species, tissue, exposure duration, and thermal conditions, with antioxidant enzyme activities showing increases, decreases, or no significant changes under different temperature exposures [65]. Similar findings were reported in the predatory mite *Neoseiulus barkeri*, where short-term heat stress significantly stimulated catalase activity, particularly at moderate to high temperatures [66]. Studies in broiler chickens have also shown that heat stress is related to oxidative stress and changes in the antioxidant defense system, and that antioxidant supplementation can support antioxidant enzyme activity during heat stress conditions [67,68].

As for vitamin E, vitamin E levels increased with increasing environmental temperature and tended to show an inverse relationship with total antioxidant capacity, indicating that the intracellular antioxidant system may not respond entirely in the same direction. Vitamin E is a lipid-soluble antioxidant that plays an important role in preventing lipid peroxidation by inhibiting the continuation of lipid radical chain reactions and reducing damage to polyunsaturated fatty acid-rich cell membranes [69]. Redox protection systems involving vitamin E and coenzyme Q also contribute to maintaining antioxidant protection within cell membranes and limiting lipid peroxidation, a key oxidative process implicated in ferroptosis [70]. Therefore, the increase in vitamin E at elevated temperatures may reflect a compensatory cellular response that limits lipid peroxidation and protects cell membranes from oxidative damage.

Malondialdehyde levels measured using the TBARS method in butterfly lizards peaked at 37 °C, while protein oxidation levels tended to increase with temperature, reaching their highest levels within the 35–39 °C range. These findings suggest that, under elevated environmental temperatures, butterfly lizard blood cells accumulate greater oxidative damage to lipids and intracellular proteins. Generally, ROS can promote lipid peroxidation, resulting in the formation of thiobarbituric acid-reactive products, including MDA [10]. Therefore, the increased TBARS/MDA values observed in the present study are consistent with increased lipid peroxidation under elevated temperature conditions. Increased protein oxidation also indicates the accumulation of oxidative damage to intracellular protein components. As temperature increases, ROS can react with sulfhydryl groups and amino acid side chains, resulting in changes in protein structure, such as protein carbonylation, polypeptide chain breakage, and protein aggregation [71]. Comparable findings were reported by Gilbert et al. [72] in the lizard *Gallotia galloti*, where protein carbonylation and oxidative stress-related protein modifications were associated with the environmental conditions and temperature ranges experienced by animals in nature. However, because the TBARS assay is not entirely specific for MDA, these values should be interpreted with consideration of the inherent analytical limitations of the method.

The increase in oxidative damage is also consistent with changes in the antioxidant system observed in this study, particularly the decrease in glutathione and total antioxidant capacity, together with changes in glutathione peroxidase and catalase. This pattern may indicate that antioxidant regulation becomes altered or insufficient to fully control reactive oxygen species during progressive thermal elevation, contributing to the accumulation of oxidative intermediates and oxidative products in butterfly lizard blood cells. Red blood cell length in butterfly lizards increased with temperature. The greatest red blood cell length was observed at 37 °C, while the cell length-to-nuclear length ratio (CL/NL ratio) increased in the 35–37 °C range without changes in cell width or nuclear length. This suggests that the observed changes are more likely related to elongation of the cytoplasmic compartment rather than an increase in overall cell size. These findings may indicate that elevated ambient temperatures can affect the structure and physical properties of red blood cells. Generally, the red blood cell membrane consists of a lipid bilayer and a membrane cytoskeleton containing important proteins such as spectrin, actin, and other binding proteins that play roles in rigidity, flexibility, and the shape-shifting ability of the cell [73,74,75]. Previous studies have shown that oxidative modifications can affect membrane lipids and cytoskeletal proteins, thereby altering cellular structure and mechanical properties [76,77]. Actin is also known to be sensitive to redox modifications and plays an important role in maintaining cell shape and deformability [78,79].

One possible explanation for these morphological changes may involve temperature-dependent alterations in membrane properties or cytoskeletal organization. The spectrin–actin cytoskeleton plays an important role in maintaining erythrocyte shape and mechanical properties [76]; however, its involvement in the observed morphological changes cannot be established because cytoskeletal components were not directly examined in the present study. These findings differ from observations in juvenile red spotted grouper (*Epinephelus akaara*), in which thermal stress induced several erythrocytic morphological abnormalities and alterations in red blood cell shape [80], and from studies in rainbow trout (*Oncorhynchus mykiss*), which reported changes in red blood cell shape from oval to spherical under heat stress, reflecting alterations in red blood cell morphology and cellular architecture [81]. Such differences may be related to species variation, physiological characteristics of different ectotherms, and varying levels and durations of temperature exposure, all of which can influence physiological fitness and responses to thermal stress among animal species [82].

Overall, the findings indicate coordinated temperature-dependent changes across physiological responses, cellular metabolism, redox balance, antioxidant defenses, iron homeostasis, oxidative damage, and red blood cell morphology in butterfly lizards. Increasing temperature was accompanied by increased body temperature and cellular metabolic activity, together with temperature-dependent changes in hydrogen peroxide and antioxidant defenses, including glutathione, glutathione peroxidase, catalase, vitamin E, and total antioxidant capacity. At higher temperatures, these changes occurred alongside decreased ferrous iron, increased ferric iron, and increased TBARS/MDA values and protein oxidation, consistent with alterations in iron redox status and increased lipid and protein oxidative damage. Red blood cell morphology also changed across the temperature gradient; in particular, cell length increased significantly, whereas the CL/NL ratio showed a numerical but non-significant increase. These morphological changes may potentially involve alterations in membrane properties or cytoskeletal organization; however, these mechanisms were not directly assessed in the present study. Collectively, these findings suggest that acute thermal elevation is accompanied by coordinated changes in cellular metabolism, antioxidant regulation, iron homeostasis, oxidative status, and red blood cell morphology. However, because correlation or multivariate analyses were not performed, these relationships should be interpreted as integrated temperature-dependent patterns rather than statistically demonstrated associations among the measured variables.

This study has limitations because butterfly lizards are small, and the blood volume obtained from each individual was insufficient for comprehensive in vitro biochemical and cellular analyses. Therefore, blood samples from multiple individuals were pooled to obtain sufficient sample volume for all analyses. Because pooling was performed before the in vitro experiment, individual biological variation was not retained in the biochemical dataset. The replicate tubes represented technical/exposure replicates derived from the same pooled blood preparation. Consequently, the in vitro findings should be interpreted as temperature-dependent responses of the pooled cellular preparation under the experimental conditions rather than as estimates of variability among individual butterfly lizards. In addition, the present study did not assess ferroptosis-specific molecular or functional markers; therefore, the observed changes in iron homeostasis, antioxidant defense, and lipid oxidation should not be interpreted as direct evidence of ferroptosis. Further studies using independent biological samples, together with ferroptosis-specific markers and functional validation, are required to determine whether ferroptotic mechanisms contribute to heat-induced cellular injury and to allow formal evaluation of relationships among physiological and cellular variables using correlation or multivariate analyses.

## 5. Conclusions

Acute environmental temperature elevation in butterfly lizards (*Leiolepis belliana belliana*) was associated with increased body temperature, while the pooled blood-cell preparation showed increased cellular metabolic activity and temperature-dependent changes in oxidative status, antioxidant defenses, and iron homeostasis. Increased cellular metabolic activity may reflect altered cellular reducing activity and metabolic responses to acute thermal elevation, occurring alongside reactive oxygen species production and subsequent oxidative imbalance. Changes in malondialdehyde and protein oxidation, together with decreased total antioxidant capacity and alterations in ferrous and ferric iron, indicate disruption of oxidative balance and possible changes in iron redox status during acute temperature elevation. Simultaneously, the antioxidant system of the blood-cell preparation responded through alterations in glutathione, glutathione peroxidase, catalase, and vitamin E. At higher temperatures, increased oxidative damage was accompanied by changes in red blood cell morphology, particularly increased cell length. Collectively, these findings demonstrate temperature-dependent physiological responses in butterfly lizards and coordinated biochemical and morphological changes in the pooled blood-cell preparation. However, these cellular findings should be interpreted within the context of the pooled experimental preparation and should not be considered direct evidence of ferroptosis. Further studies using independent biological samples and ferroptosis-specific molecular and functional markers are required to determine the broader biological significance and underlying mechanisms of these responses.

## Figures and Tables

**Table 1:** Effects of acute thermal exposure on red blood cell morphometry in butterfly lizards (*Leiolepis belliana belliana*).

Parameters	Environmental Temperature (°C)						*p*-Value
	27 °C	29 °C	31 °C	33 °C	35 °C	37 °C	
1. CL (µm)	15.49 ± 0.34 d	16.54 ± 0.16 cd	17.51 ± 0.62 bcd	18.54 ± 0.40 bc	19.56 ± 0.43 ab	20.61 ± 0.25 a	0.0005
2. CW (µm)	10.76 ± 0.83	11.12 ± 0.81	11.18 ± 1.80	10.97 ± 0.19	10.86 ± 0.90	11.59 ± 1.29	0.7898
3. NL (µm)	6.61 ± 0.32	6.45 ± 1.05	6.77 ± 0.79	6.60 ± 0.38	6.14 ± 0.34	6.53 ± 0.18	0.6800
4. NW (µm)	4.72 ± 0.41	4.75 ± 1.05	4.79 ± 1.08	4.75 ± 0.10	4.31 ± 0.14	4.33 ± 0.51	0.8814
5. CL/CW ratio	1.44 ± 0.11	1.49 ± 0.12	1.60 ± 0.28	1.69 ± 0.05	1.81 ± 0.12	1.79 ± 0.17	0.2060
6. NL/NW ratio	1.41 ± 0.08	1.37 ± 0.07	1.44 ± 0.22	1.39 ± 0.09	1.43 ± 0.12	1.52 ± 0.14	0.5273
7. CL/NL ratio	2.34 ± 0.08	2.61 ± 0.42	2.61 ± 0.35	2.82 ± 0.17	3.19 ± 0.11	3.16 ± 0.06	0.1054
8. CW/NW ratio	2.28 ± 0.04	2.39 ± 0.40	2.36 ± 0.28	2.31 ± 0.05	2.52 ± 0.26	2.68 ± 0.14	0.3639

Different letters indicate significant differences among temperatures (*p* < 0.05). Data are presented as mean ± SD. CL = red blood cell length; CW = red blood cell width; NL = red blood cell nucleus length; NW = red blood cell nucleus width.

## Data Availability

The raw data supporting the conclusions of this article will be made available by the authors on request.

## Supplementary Materials

The following supporting information can be downloaded at https://www.mdpi.com/article/10.3390/ani16172776/s1. Table S1: Morphometric measurements and body weights of butterfly lizards (*Leiolepis belliana belliana*) used in the study.
