# Supplementary material for: Acute Thermal Elevation Alters Cellular Metabolic Activity, Redox Balance, and Iron Homeostasis in Butterfly Lizards (Leiolepis belliana belliana)
Source: Animals (Basel). 2026 Sep 3;16(17):2776. doi: 10.3390/ani16172776 (PMC13564942; doi:10.3390/ani16172776)

Table S1. Morphometric measurements and body weights of butterfly lizards (*Leiolepis belliana belliana*) used in the study. Head length, trunk length, tail length, and chest circumference are expressed in centimeters (cm), and body weight is expressed in grams (g). The anatomical locations used for morphometric measurements are illustrated below the table.

Animal ID	Head length (cm)	Trunk length (cm)	Tail length (cm)	Chest circumference (cm)	Body weight (g)
A	3.5	7.5	23.5	5.2	30.0
B	4.3	7.5	22.5	7.0	32.0
C	4.0	7.3	22.7	7.0	37.0
D	3.4	6.2	22.3	5.5	32.0
E	3.3	8.0	23.4	6.5	45.0
F	3.5	7.7	22.2	7.0	44.0
G	3.5	7.5	22.4	6.3	41.0
H	3.5	6.7	23.0	5.6	32.0
I	4.0	8.0	23.3	7.0	50.0
J	3.0	6.2	21.1	5.0	36.0

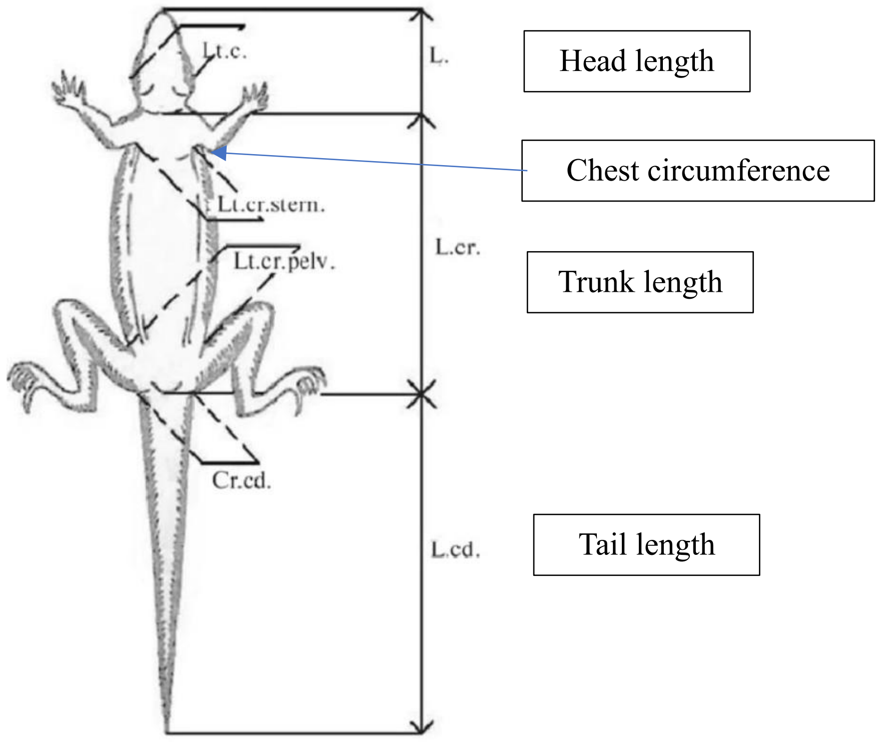

Supplement: Supplementary file 1 [file animals-16-02776-s001.zip › animals-4443614-supplementary.pdf]
